# Improving phosphate use efficiency in the aquatic crop watercress (*Nasturtium officinale*)

**DOI:** 10.1093/hr/uhac011

**Published:** 2022-02-11

**Authors:** Lauren Hibbert, Gail Taylor

**Affiliations:** School of Biological Sciences, University of Southampton, Southampton, Hampshire, SO17 1BJ, UK; Department of Plant Sciences, UC Davis, Davis, CA, 95616, USA; School of Biological Sciences, University of Southampton, Southampton, Hampshire, SO17 1BJ, UK; Department of Plant Sciences, UC Davis, Davis, CA, 95616, USA

## Abstract

Watercress is a nutrient-dense leafy green crop, traditionally grown in aquatic outdoor systems and increasingly seen as well-suited for indoor hydroponic systems. However, there is concern that this crop has a detrimental impact on the environment through direct phosphate additions causing environmental pollution. Phosphate-based fertilisers are supplied to enhance crop yield, but their use may contribute to eutrophication of waterways downstream of traditional watercress farms. One option is to develop a more phosphate use efficient (PUE) crop. This review identifies the key traits for this aquatic crop (the ideotype), for future selection, marker development and breeding. Traits identified as important for PUE are (i) increased root surface area through prolific root branching and adventitious root formation, (ii) aerenchyma formation and root hair growth. Functional genomic traits for improved PUE are (iii) efficacious phosphate remobilisation and scavenging strategies and (iv) the use of alternative metabolic pathways. Key genomic targets for this aquatic crop are identified as: *PHT* phosphate transporter genes, global transcriptional regulators such as those of the *SPX* family and genes involved in galactolipid and sulfolipid biosynthesis such as *MGD2/3, PECP1, PSR2, PLDζ1/2* and *SQD2.* Breeding for enhanced PUE in watercress will be accelerated by improved molecular genetic resources such as a full reference genome sequence that is currently in development.

## Introduction

### Watercress – An aquatic leafy-green crop

**Figure 1:**
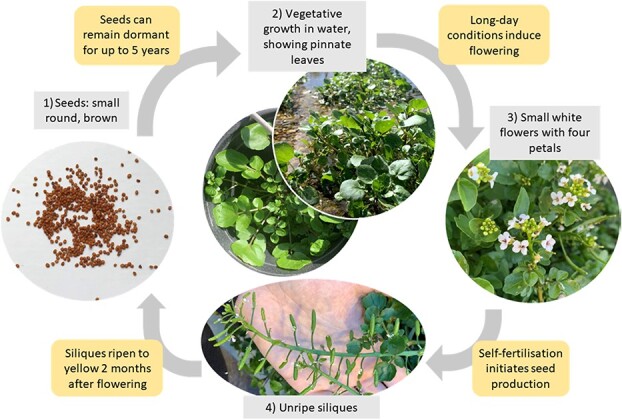
**Watercress life cycle.** Plants with characteristic pinnate compound leaves, hollow floating stems and numerous adventitious roots grow in flowing water. They begin to flower under long-day conditions in May, producing small white flowers with four petals. Siliques develop and ripen approximately two months after flowering, each containing four rows of small, round seeds.

Watercress (*Nasturtium officinale* R. Br.) is a semi-aquatic plant that grows in flowing shallow freshwater and is found across Europe, Asia, the Americas, the Caribbean, New Zealand and Australia (Fig. 1) [1]. Watercress is placed within the Brassicaceae family together with several other important food crops including broccoli, kale, cabbage, and mustard.

A significant amount of commercial aquatic watercress cultivation is centred in a few locations including Florida in the USA, southern Spain and Portugal, France and the south of England, with 90% of production occurring in Dorset, Hampshire and Wiltshire [2]. These chalky areas provide nutrient-rich spring water and boreholes that directly supply the watercress beds [3].

Phosphate-rich fertiliser is used to boost crop yield; however, this presents a major challenge in watercress production since it results in direct leakage of phosphate into the waterways which have high conservation value. Excess phosphate results in eutrophication of aquatic ecosystems, a process where nutrient enrichment of water sources results in excessive algal and plant growth, and subsequent disruption of ecosystem community dynamic [4]. Approximately 90% of watercress farms in the UK are on, or upstream of, a Site of Special Scientific Interest (SSSI), increasing the pressure to minimise phosphate release [5].

#### Phosphate and the environment

Phosphate (P) is vital for plant survival; it forms the phosphodiester bonds that link nucleotides in nucleic acids and is critical for the structure of proteins and carbohydrate polymers, for powering cells through the release of phosphate from ATP and for regulating several metabolic pathways [6, 7]. Symptoms of P deficiency are retarded growth, increased root:shoot biomass, decreased leaf area and often dark green or purple-red colouration in severely deficient plants due to anthocyanin production [8–11].

Ninety percent of the global demand for phosphorus is used for food production, however, rock phosphate is a limited resource with estimates that reserves could be exhausted in the next 50–100 years [12–14]. In addition, most of the remaining rock phosphate reserves areunder the control of only a few countries, with Morocco and the Western Sahara holding over 70% of the total reserves, making it sensitive to political instability [15, 16]. This, combined with increasing costs of extraction and issues of eutrophication, make reducing fertiliser use an important global driver [17–19]. The high reactivity and low solubility of phosphates make them commonly the growth-limiting nutrient for plants. Accounting for fertiliser application, approximately 30% of global cropland area exhibits soil P deficits although global P imbalances in water sources have not been investigated to any significant extent [20].

#### Phosphorus in the soil

Phosphates in soils are classified into inorganic P (Pi) and organic P (Po) and fractionised based on their solubility [21, 22]. Po typically comprises 30–70% of total soil P, however this is highly variable with values up to 90% reported [23–26]. Pi derived from rocks is only present in poorly soluble forms and any soluble phosphates released in the soil are rapidly immobilised into insoluble forms by interacting with adsorbing agents such as Al and Fe oxides or Ca and Mg carbonates, such that only a small amount of P in soils is accessible to plants (primarily in the form of inorganic orthophosphate compounds such as H_2_PO_4_^−^, HPO_4_^2−^ and PO^3−4^) [13]. The availability of P is dynamic, changing over time depending on various factors such as temperature, water content and soil pH [27–29].

#### Phosphorus in aquatic systems and potential for eutrophication

**Figure 2:**
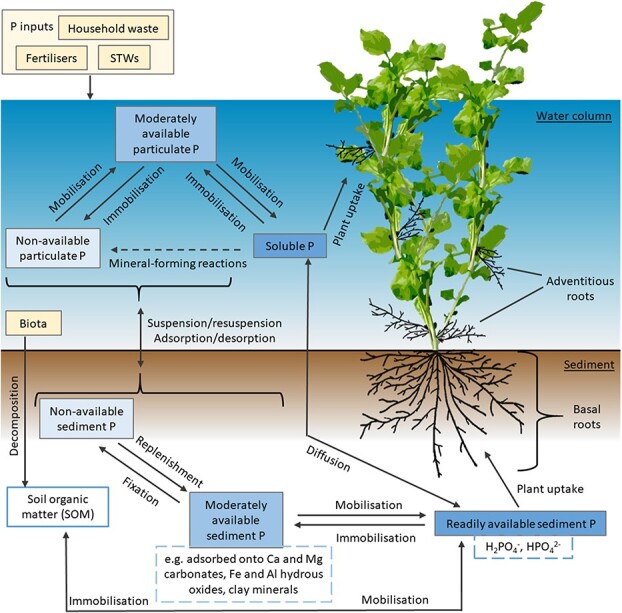
**Cycling of different phosphate (P) fractions in natural aquatic environments.** Readily available P (in the form of H_2_PO_4_^−^ and HPO_4_^2−^ ions) is taken up in the sediment by basal roots and in the water column by adventitious roots. STWs = sewage treatment works.

Like soil, P in aquatic systems is also divided into different fractions based on solubility and reactivity in aquatic systems, with dissolved orthophosphate the most bioavailable [30, 31]. P in water adsorbs to oxides and tightly binds with carbonates in the same manner as when in soil. However, the P inputs to natural water systems and the interaction with P in bed sediments is altered. This creates a dynamic source of phosphorus that transfers between particulate and dissolved forms, between bed sediments and the water column, and between dead and living material (Fig. 2). In a watercress bed, the sediment is shallow gravel and thus P uptake from water likely represents the major P source. This is reflected in a study by Cumbus and Robinson (1977) who found that a greater proportion of P was absorbed by the adventitious roots of watercress (in the water), compared to basal roots (in the sediment) [32]. However, some organic detritus held within the sediment should still be considered. Phosphate dynamics in hydroponic agricultural systems such as watercress beds have not been studied, representing a knowledge gap, but P is likely uniformly distributed due to flowing water and regular maintenance of P concentrations.

Since P retention in sediments is high, P delivery into freshwater systems is largely governed by release from point sources such as sewage treatment works (STWs), leaking septic tanks, and from excess fertiliser application [33–35]. Globally, domestic sources contribute 54% to total P inputs into freshwater systems, 38% from agriculture and 8% from industry [36]. Although substantial steps towards P reduction in freshwaters have been made over the last 50 years there is still much to be done, with only 40% of European surface waters currently in good ecological status [37, 38]. Eutrophication of watercourses is also prevalent across the UK: in the most recent analysis, 55% of river water bodies in England failed to meet the revised P standards for good ecological status [38]. Eutrophication is both an economic as well as environmental issue. In the US, the economic damage of eutrophication equates to $2.2 billion (~£1.6 billion) annually, due to losses in recreational water use, waterfront real estate, recovery of endangered species and drinking water [39].

Naturally, phosphate levels in chalk aquifers (where watercress farms are typically found) are less than 20 μg/l, however, inputs of phosphate rapidly increase these concentrations above P targets downstream of watercress farms [2, 5]. In the river Itchen (Hampshire, UK) where several watercress farms are located, total SRP load comes predominantly from sewage treatment works (84.2%) but watercress beds can be responsible for up to 62% of the total reactive phosphate in some chalk streams, suggesting room for improvement in P management [5]. Casey and Smith (1994) found watercress beds increased mean P concentrations which may cause undesirable growth of algae and disruption of community dynamics [2].

One important strategy to tackle this problem of eutrophication is through plant breeding. By breeding watercress varieties with improved phosphorus use efficiency (PUE), the impact of watercress farming on eutrophication could be minimised. To date, no breeding for nutrient use has been conducted in watercress even though P release represents a clear issue in watercress production.

#### Improving the environmental footprint of watercress through enhanced PUE

Phosphate use efficiency (PUE) is defined as the capacity for biomass production using the P absorbed [40, 41]. Here PUE is used as a broader term that also encompasses phosphate acquisition efficiency, defined as the ability to take up P, as has been used in several studies [42–45]. Plant traits underpinning PUE can be observed at the macroscopic, microscopic, and molecular levels and we consider their relevance to future breeding for enhanced PUE.

To date, knowledge on P acquisition by aquatic plants only covers the effectiveness of plants for phytoremediation (removal of toxic contaminants by plants), rather than breeding for PUE in aquatic crops such as water chestnut (Eleocharis dulcis), water spinach (Ipomoea aquatica), lotus (Nelumbo nucifera) and watercress. Present information does not cover morphological or genetic components to improve PUE in aquatic species, and with new plant species emerging as suggested model organisms, watercress is offered as a model crop for aquatic systems [46–48]. The need for aquatic model crops is only exacerbated by increasing market value in indoor hydroponic cultivation systems [49].

#### Traits for improving P acquisition

##### Macroscopic traits: Root structural architecture (RSA)

Root structural architecture (RSA) defines the spatial configuration of the root system and variation in RSA can reflect efficient phosphate uptake by plants [50]. Much of the current literature focuses on RSA traits in maize (*Zea mays*), common bean (*Phaseolus vulgaris*) and *Arabidopsis thaliana* with RSA shown to be a highly plastic trait, changing in response to the availability of water, nutrients and hormone signalling [51–53]. For example, ethylene is involved in the promotion of lateral root growth, root hair growth and inhibition of primary root growth under low P conditions [54].

*Reduced root growth angle (RGA):* is one of the most important RSA traits for improving P acquisition in many soil-grown species. In maize and common bean, lower RGA (shallow roots) is associated with increased P accumulation and improved growth in P deficient soil where P is concentrated in the topsoil [55–57]. However, in aquatic systems when P is likely homogenously distributed, a lower RGA is unlikely to be advantageous. Root growth angle therefore is not considered important as a trait for enhance PUE in aquatic plants.

*Increased lateral root density:* (arising from the pericycle of mature roots) also enhances P acquisition by allowing plants to explore outside P-depleted zones. Using maize recombinant inbred lines (RILs) with contrasting lateral rooting phenotypes, significant differences in phosphorus acquisition, biomass accumulation and relative growth rate were observed under low phosphorus availability [58, 59]. Increased investment in the production of lateral roots was shown to be cost-effective under low P. For aquatic crops, enhanced lateral root density is an important trait for enhanced PUE since it increases root surface area for P uptake.

*Adventitious roots:* from above ground structures can enhance topsoil foraging by up to 10% in stratified soil [60]. These require a lower metabolic investment than basal roots, however, in uniform soil they can limit P acquisition by hindering the growth of basal roots [61]. In aquatic and flooding-tolerant plants, adventitious roots are important for P uptake within the water column [62, 63]. There is positive correlation between the size of the adventitious root system and P uptake in bittersweet (*Solanum dulcamara*) plants under long-term submergence, and as mentioned, adventitious roots are responsible for a higher proportion of P acquisition in watercress than basal roots [32, 64]. Thus, in an aquatic crop such as watercress, increased adventitious root production is a key trait for enhanced PUE.

##### Microscopic traits

*Root cortical aerenchyma*: (RCA; enlarged spaces in the root cortex) are an adaptation to waterlogged soils and reduce the risk of asphyxiation and their formation has been suggested to increase P uptake in deficient soils [65]. Several studies have reported increase in RCA formation in maize under these conditions [66–68]. Modelling of root architecture in maize and bean has shown RCA may increase the growth of plants up to 70% in maize and 14% in bean under low P availability largely due to remobilisation of P from dying cells [67]. However, formation of aerenchyma is also associated with reduced root hydraulic conductivity in maize which may impede transport of water, but is unlikely to be relevant to hydroponically grown crops [69]. Most aquatic plants, including watercress, form aerenchyma constitutively in roots and stems to aid internal gas exchange and maintain strength under water pressure [70]. For aquatic crops such as watercress, formation of root aerenchyma is an important trait for selection for enhanced PUE.

*Root hair density and root hair length:* root hair density increases up to 5 times in low P conditions [71–73]. Using Arabidopsis mutants, Bates & Lynch (1996) found hairless plants had lower biomass and produced less seed than wild-type plants at low phosphorus availability [71]. Root hairs increased root surface area by 2.5 to 3.5-fold in barley and wheat (Triticum aestivum), respectively, and there was an almost perfect correlation between P uptake and root hair surface area [74]. Root hair traits vary substantially between genotypes and the genetic control underlying their formation is well understood, thus making them an excellent target for plant breeding programs [75–78]. Root hair length and density are likely to be important for PUE in aquatic crops as they significantly increase root surface area for P uptake.

Synergism of root phenotypes should also be considered. A modelling approach in Arabidopsis showed that the combined effects of root hair length, root hair density, tip to first root hair distance and number of trichoblast files (responsible for forming root hairs) on P acquisition was 3.7-fold greater than their additive effects [72].

For aquatic species such as watercress, the root ideotype for determining optimal P acquisition remains unknown. Although, absorption of P through the shoots is still debated, root uptake is generally regarded as the mode of P uptake in aquatic plants [79–81]. Watercress beds have a fine gravel substrate which contains negligible amounts of P [82]. The substrate within watercress beds is likely too shallow to allow for significant stratification of P, thus a shallower basal root angle would be unlikely to provide much adaptive benefit. In groundwater sources, P may be distributed more homogenously due to turbulent flowing water, so RGA will not assist within the water column. Nevertheless, even with homogenous P distribution, plants with shallower root systems have been shown to encounter less inter-root competition with roots on the same plant so RGA could provide an adaptive value in this sense [83]. Cumbus & Robinson (1977) studied P absorption by the adventitious and basal roots of watercress and found that the adventitious roots absorbed a higher proportion of P at low P concentrations, despite having a lower biomass compared to the basal root tissue [32]. Thus, adventitious roots are also a key trait for analysing watercress PUE. Increased production of lateral roots, adventitious roots and root hairs all increase root surface area, and thus will increase P acquisition from the water and sediment and are important traits for PUE in watercress.

In addition, the root cap can account for 20% of the phosphate absorbed by the roots of Arabidopsis [84]. Therefore, increasing the number of roots increases the number of root tips and the number of these “hot spots” for phosphate acquisition.

##### Improving P acquisition at the molecular level: P transporters, organic acid exudation, phosphatase secretion

Plants are reliant on phosphate transporters to acquire P from the environment and transport P between tissues, and this includes for aquatic plants. The PHT1 (PHOSPHATE TRANSPORTER 1) family is the most widely studied group of P transporters and is primarily responsible for P uptake but also has a role for P transport between tissues [85–87]. A broad range of expression patterns are associated with different PHT1 genes but generally, higher expression of PHT1 genes is associated with improved shoot biomass accumulation and P tolerance [88–90]***.*** Watercress with higher PHT1 expression may result in improved biomass accumulation in P deficient water, but this has yet to be tested.

Additional traits that are important in other crops are organic acid (OA) exudation and phosphatase activity. Since these control release of P from organic forms in the soil, they are less relevant to watercress cultivation where P released from bound sources would be rapidly lost to the watercourse. However, phosphatases that remobilise P from intracellular sources have been identified in Arabidopsis so similar phosphatases could enhance internal P utilisation in watercress [91, 92].

#### Traits for improving P utilisation

##### Improving utilisation at the molecular level

Alongside phosphate acquisition, PUE also refers to more efficient P utilisation associated with re-translocation and recycling of stored P, that relies on effective P transportation within the plant, P scavenging, and use of alternate biochemical pathways that bypass P use [93–95]. Re-translocation between plant tissues is governed by transporters such as PHT transporters and PHO transporters. Unlike, PHT transporters which regulate P acquisition too, PHO transporters are solely responsible for P transport into vascular tissues and cells [96]. The genetic control underlying these PUE mechanisms is covered in the subsequent section.

Alternative P use strategies includes substituting phospholipids in cell walls with sulfolipids and galactolipids. Several enzymes in the glycolytic pathway depend on P so bypass enzymes such as pyrophosphate-dependent phosphofructokinase (PPi-PFK), phosphoenolpyruvate carboxylase (PEPC) and pyruvate phosphate dikinase (PPDK) can be recruited to use pyrophosphate (PPi) for a P donor and conserve limited ATP pools [94, 95, 97, 98]. Several studies have reported increased PEPC activity under P deprivation [99, 100]. The mitochrondrial electron transport chain responds by utilising non-phosphorylative pathways [101]. Acid phosphatases (APases) in intracellular (vacuolar or cytoplasmic) spaces or present in the apoplast can increase P availability by remobilising P from senescent tissues and the extracellular matrix [102].

Both aspects of PUE (acquisition and utilisation) rely on accurate sensing of the P state within the plant and external environment to alter global gene expression and ensure appropriate responses to upregulate P uptake and P use pathways [54, 103].

#### Understanding the genetic control of PUE in watercress

Many agricultural traits, such as PUE, are under the control of multiple rather than single genes, defined as quantitative traits and can be mapped on the genome as quantitative trait loci (QTL) and used to identify co-located candidate genes [104]. QTL mapping and candidate gene mining are well-established techniques and linking phenotype to genotype in this way provides understanding of the genetic control of PUE traits of interest. These genomic loci can then be used to develop individual molecular markers for breeding or in genomic selection strategies that utilise multiple marker data.

##### QTL for PUE

QTL for overall PUE metrics as well as QTL for more specific architectural root traits associated with low P tolerance have been identified in several economically important crops including soybean, soybean (Glycine max), rice (Oryza sativa), maize and common bean [105–109]. RSA is extremely plastic, subject to effects of hormone signalling, environmental stimuli and under the control of several genes so elucidating these QTL is challenging [110, 111].

Studies on other Brassicaceae species are likely of most genetic relevance for QTL mapping in watercress, however QTL associated with other species such as soybean, rice, sorghum and wheat are summarised in Table 1. P-starved Arabidopsis exhibit longer root hairs and higher root hair density, decreased primary root length and increased lateral root density [112, 113]. Three QTL, (including one QTL later identified as the gene *LPR1*) were identified which explained 52% of the variance in primary root length [114]. In rapeseed (*Brassica napus*) primary root length decreases, lateral root length and density increases with declining P concentration [115]. Several QTL are associated with these changes and many co-locate with QTL for root traits in Arabidopsis. A more recent study used over 13 000 SNP markers to construct a genetic linkage map in rapeseed, where 131 QTL were identified in total across different growth systems and P availabilities [116]. However, only four QTL were common to all conditions, demonstrating strong environmental effects determining these QTL. To date, there is no published literature on QTL associated with aerenchyma formation under low P in any plant species and no studies exist on QTL mapping for root traits in watercress. Identification of QTL and markers associated with PUE could accelerate breeding for nutrient use and reduce the environmental impact associated with watercress cultivation.

**Table 1:** **A selection of studies identifying QTL associated with various PUE traits in terrestrial species.** No studies exist for QTL mapping of PUE in aquatic crops but where studies include hydroponic growth systems this is noted

Species	QTL identified	Details	Reference
*Arabidopsis thaliana*	Three QTL for primary root length	*LPR1*: explained 52% of variance in primary root length (PRL). 2^nd^ QTL (*LPR2*) for primary root cell elongation under low P. 3^rd^ QTL (*LPR3*) associated with PRL regardless of P status.	[114]
*A. thaliana*	24 QTL for leaf Pi concentration	Identified 152 genes associated with leaf anion concentration under different P conditions.	[117]
Barley	17 QTL for PUE, yield and phosphate acquisition efficiency	QTL explained 11–24.7% of variation. 14 candidate genes for P efficiency identified from these QTL.	[118]
rapeseed	131 QTL across different environments and P conditions – 4 common to all	Associated with numerous root growth and yield traits. 1 major QTL explaining 18% of variation in lateral root density. Hydroponic growth systems were included.	[116]
*rapeseed*	38 QTL for RSA and biomass traits	3 loci accounted for 27.9% of variation in primary root length at low P. Many QTL co-locate with biomass QTL in other studies.	[115]
*rapeseed*	71 QTL total for traits including biomass and measures of PUE	28 QTL were specific to low P conditions. Hydroponically grown so of interest for aquatic crops.	[119]
Bean	16 QTL for RGA	Associated with gravitropic root traits but together only explain 15.9% of total variability in RGA	[120]
Bean	19 QTL for adventitious root formation	Two of the 19 QTL accounted for 61% of variation in adventitious root formation under low P. Includes hydroponic environment.	[121]
Rice	16 QTL for several P tolerance traits	Associated with numerous traits including biomass, root:shoot ratio, root volume, P content in seed. Including a QTL hotspot of 10 QTL, 5 of which were major QTL.	[122]
Rice	*Pup1* – root growth and P tolerance	Identification of *PSTOL1* gene associated with this QTL. Overexpressor *PSTOL1* plants had enhanced root growth, P uptake and up to 60% higher grain yield in P-deficient conditions. Included study in hydroponic systems.	[123–125]
Rice	18 QTL for PUE and root:shoot ratio	One common QTL for 3 traits (*qRPUUE9.16*): phosphorus use, relative physiological phosphate use efficiency, and relative phosphate uptake efficiency. 4 candidate genes located on this QTL.	[126]
Rice	21 QTL associated with plant growth inhibition under P deprivation	158 genes co-located with QTL e.g. *GLYCEROPHOSPHODIESTER PHOSPHODIESTERASE 13*, involved in Pi transport	[127]
Sorghum	14 QTL for grain yield/root morphology	Grain yield QTL linked to three root morphology QTL. These QTL are tightly linked and near homologs of rice *PSTOL1* gene. Hydroponics used for root phenotyping.	[128]
Soybean	172 QTL (3 major) for P use and photosynthetically related traits under P stress	3 major QTL (*q14–2*, *q15–2*, and *q19–2*). *q14–2* for root dry weight and P uptake is underlined by the gene *GmETO1*. Included experiments using hydroponics.	[129 ,130]

##### Candidate genes for PUE

Genes involved in phosphate acquisition and utilisation identified in other species for PUE are likely to be candidate genes in aquatic crops like watercress. A set of 95 core phosphate starvation-inducible genes, whose expression changes >2 fold under P deficiency have been identified in Arabidopsis, resulting in the identification of candidate genes for PUE in several other crops including wheat, maize, oat (Avena sativa) rice and white lupin (Lupinus albus) under P deficiency [131–136]. The Arabidopsis “phosphatome” contains a large portion of genes whose function have not been characterised under P deprivation, and thus many of these are not discussed here. A selection of the most important PUE genes are summarised in Table 2.

**Table 2:** **Candidate genes for PUE breeding in watercress** categorised based on function under P deprivation *Additional key transcriptional regulators are multifunctional, with roles in RSA determination and control of other P responses. Genes are those in *Arabidopsis thaliana* (*At*) unless specified otherwise (e.g. *Os* = rice). Those in bold are major components of the P response whose expression is induced more than tenfold in at least 2 independent studies, as found by Lan *et al.* (2015) and are considered as key targets for PUE in watercress. P- refers to P deficient conditions and P+ represents P-sufficient conditions.; WT = wild type; OE = overexpression; PSR = phosphate starvation responses

Candidate gene	Function for PUE	Mutant phenotypes	References
P-responsive root development:			
*PHOSPHATE DEFICICENCY RESPONSE 2* (*PDR2*)	● Involved in gene expression changes in the plant apical meristem under P-. ● Monitors environmental P status. ● Functions together with *LPR1.*	*Pdr2* in P-: ● Shorter primary root ● Reduced P acquisition	*At:* [ 139, 174]
*LOW PHOSPHATE ROOT 1 (LPR1)*	● Functions together with *LPR2* and *PDR2* to alter root meristem activity. ● Implicated in auxin responses to P starvation-mediated RSA changes.	*Lpr1* in P-: ● Decreased root hair density ● Increased primary root length ● Higher P uptake.	*At:* [140, 174, 175]
*PHOSPHATE DEFICIENCY ROOT HAIR DEFECTIVE1* (*PER1*)	● Role in signalling during root development.	*Per1* in P-: ● 60% reduction root hair length ● No significant differences in P content	*At:* [176]
*TRANSPORT INHIBITOR RESPONSE1 (TIR1)*	● Involved in the lateral root response by increasing degradation of transcriptional repressors of auxin genes (AUX/IAA proteins) in P-	*Tir1* in P-: ● Impaired in lateral root formation = reduced root surface area and P uptake.	*At:* [149]
*AUXIN RESPONSE FACTOR 19* (*ARF19*)	● Regulates genes involved in root hair elongation.	*Arf19* in P-: ● Reduced root hair elongation	*At:* [148]
*ROOT HAIR* *DEFECTIVE 6-LIKE 2* (*RSL2*) and *ROOT HAIR DEFECTIVE 6-* *LIKE 4* (*RSL4*)	● Involved in root hair formation in P-	*Rsl2/6* in P-: ● Reduced root hair elongation	*At:* [148, 177, 178]
*OsPTF1*	Rice transcription factor induced in P-. ● Involved in root growth.	Rice OE in P-: ● Increased root and shoot biomass and P content .by ~30% ● Enhances tolerance to P-deficiency by improving P uptake.	Rice: [179]
*PHOSPHORUS-STARVATION TOLERANCE 1* (Os*PSTOL1*)	● Enhances early root growth in rice.	Rice OE in P-: ● Increased root dry weight, root surface area and total root length = increased rice grain yield by 60%.	Rice: [124, 180]
P transport (uptake and translocation):			
*PHT* (*PHOSPHATE TRANSPORTER*) genes	● Responsible for uptake of P via the roots and transport between tissues.	OE in P-: ● Increases P uptake, shoot P content and biomass in rice and Arabidopsis*.* Effects dependent on individual *PHT* genes.	*At*: [153, 181–183] Soybean: [151, 184] Maize: [86, 185] Rice: [186–190] Rapeseed [154, 191]
*PHO* genes (*PHO1, PHO2, PHO3*)	*PHO:* controls P efflux out of cells into xylem and acquisition of P into cells *PHO2*: regulates translocation of P from shoots to roots.	*Pho1* in P-: ● Severe shoot P deficiency with stunted growth due to deficient P loading into the xylem. *Pho2*: ● Unable to remobilise P within leaves and accumulate excessive P in shoots (in both P-sufficient and P-deficient conditions) due to the reduced ability to translocate P from shoots to roots. ● P toxicity in P+	*At*: [85, 96, 142, 150, 156, 192–194] Soybean: [195] Maize: [196] Rice: [197]
*PHOSPHATE TRANSPORTER TRAFFIC FACILITATOR* (*PHF1*)	Codes for an accessory protein for P uptake. ● Involved in trafficking of PHT1 transporter to the plasma membrane.	*Phf1*: ● Impaired in P uptake = constitutively display PSR.	*At*: [198]
Key multi-functional transcriptional regulators: *			
*ZINC FINGER OF* ARABIDOPSIS *6* (*ZAT6*)	● Regulates root development and PSR (e.g. anthocyanin accumulation)	OE in P+: ● Increased root:shoot biomass and lateral root length ● Decrease in primary root length and number of lateral roots. ● Overall significant increase in P uptake attributed to increase root surface area.	*At*: [199, 200]
*WRKY75*	● Regulates several P starvation-induced genes (e.g. phosphatases and P transporters). ● Regulates root architecture independent of P supply. ● May have mutually synergistic effects with *ZAT6*.	*Wrky75*: ● Reduced P uptake (due to reduced expression of *PHT* transporter genes) ● More susceptible to P stress.	*At*: [199]
*WRKY6 and WRKY42*	● Negative regulators of PSR via repression of *PHO1.*	● *WRKY6* overexpressors: reduced P content and increased anthocyanin content	*At:* [201]
*HPS1 (HYPERSENSITIVE TO PHOSPHATE STARVATION 1)*	● Involved in sugar-mediated responses to P- by interaction with *SUC2* sucrose transporter gene (in vascular tissues).	● *Hps1* in P+: enhances PSR: increased expression of P transporters, overproduction of APases (ACP5) and anthocyanins, shift in allocation of P from shoots to roots, shorter lateral roots and root hairs.	*At:* [146]
*PHR1 (PHOSPHATE STARVATION RESPONSE 1) and PHL1 (PHR-LIKE 1)*	Global regulators of several Pi-deficiency-responsive genes. ● Involved in transducing low P signals. ● Bind to the P1BS element in several P starvation genes.	*Phr1/phr-like 1* in P-: ● Altered P allocation between root and shoots ● Accumulate less sugar and starch ● Impaired induction of several P starvation genes	*At:* [141, 202]
*SIZ1*	Encodes a SUMO E3 ligase that is a negative regulator of P starvation-dependent signalling. ● Dual role in regulation of *PHR1* and expression of several PSR genes ● Alters RSA through control of auxin patterning	*Siz1* in P-: ● Enhanced PSR: reduction in primary root elongation, increased root:shoot biomass and root hair number and length.	*At:* [203, 204]
*SPX1, SPX2, SPX3, SPX4*	● Negative regulators of PSR genes and have a complex P-dependent inhibitory effect on PHR1.	*Spx1* and *spx2* in P-: no significant differences in P content *Spx3:* lower P tolerance, reduced root systems and impaired shoot growth	*At:* [143, 205] Rice: [144, 206, 207]
*ACTIN-RELATED PROTEIN6* (*ARP6*)	● Role for modulating responses to P- via chromatin remodelling.	*Arp6* in P+: ● Enhanced PSR ● Decreased P content	*At:* [208]
*BHLH32*	● Negative regulator of PPCK expression (involved in P scavenging), root hair formation and anthocyanin production.	*Bhlh32*in P+: ● Enhanced PSR: increased root hair length, P content and anthocyanin accumulation.	*At:* [209]
*ETHYLENE RESPONSE FACTOR070* (*ERF070*)	● Negative regulator of P homeostasis and root growth traits.	*Erf070* in P-: ● Increased number and length of lateral roots, root hair number and P content	*At:* [210]
*MYB62*	● Roles in RSA development, P uptake and acid phosphatase activity via gibberellic acid metabolism and signalling.	Overexpression of *MYB62*: ● Decrease in lateral root length in P+/P-, larger root:shoot ratio under P+. ● Increased P uptake in P+ ● Increased anthocyanin accumulation ● Suppression of several PSR genes	*At:* [211]
*At4/IPS*	● Noncoding RNAs that inhibit cleavage of miR399b on *PHO2* mRNA. Affects P allocation between root and shoot	Overexpression of AT4/IPS: reduced P content due to increased *PHO2* expression *At4*: fails to redistribute P so higher shoot P accumulation	[150 , 212]
*OsARF16*	● Role in PSR and auxin responses to root development.	*Osarf16* in P-: ● Insensitive to P deficiency ● Lower root:shoot ratio, lower root hair length and number of lateral roots ● Decreased expression of several PSR genes	Rice: [213]
*OsARF12*	● Functions in P homeostasis	*Osarf* mutants in P+/P-: ● Increased P concentration, upregulation of P transporter genes and APase activity, decreased number of lateral roots. ● Many other P responsive genes upregulated: e,g. *OsSPX1, OsSQD2, OsTIR1.*	Rice: [214]
P utilisation (P scavenging and P-bypass enzymes):			
*PAP26*	The predominant intracellular purple acid phosphatase in Arabidopsis*.* ● Recycles P from intracellular P metabolites in P- ● Expression upregulated 2-fold in P- ● Assists with P remobilisation during leaf senescence.	*Pap26* in P-: ● Reduced intracellular APase activity ● 30% decrease in shoot fresh weight ● 50% reduction in total P content in leaves	*At*: [157, 158, 215]
*PPC1, PPC2, PPC3,*	Encode Arabidopsis PEPCs. ● Provide a metabolic bypass to ensure continued pyruvate supply to tricarboxylic acid cycle in P-	*Ppc1/2/3* in P+: ● Reduced shoot biomass	*At*: [99, 160] *Brassica* spp.: [97, 100]
*PPCK1, PPCK2*	● Regulate PEPC activity by increasing PEPC phosphorylation.	*Ppck1/2* in P+: ● Reduced shoot fresh weight Effects under P- not assessed.	*At:* [99, 160, 209]
*MGD2, MGD3*	● Encode major enzymes for galactolipid biosynthesis in P- Phospholipids in cell membranes are substituted for non-phosphorus lipids (e.g. DGDG).	*Mgd2/3* in P-: ● Reduced DGDG accumulation, fresh weight, root growth and photosynthetic activity	*At:* [163]
*PHOSPHOETHANOLAMINE/PHOSPHOCHOLINEPHOSPHATASE 1 (PECP1)*	● Encodes a phosphatase that assists in the liberation of P from phospholipids.	*Pepc1* in P-: ● Shorter primary root ● No significant differences in free P content.	*At*: [165, 166, 169]
*PHOSPHATE STARVATION-INDUCED GENE 2 (PSR2)*	● Involved in the galactolipid biosynthetic process with *PECP1.* ● Assists in the liberation of P under limiting conditions.	Double mutant with *pecp1* in P-: ● No altered growth phenotype ● Reduction in choline (involved in galactolipid biosynthesis) content.	*At*: [168, 169]
*PLDZETA1* and *PLDZETA2*	● Hydrolyse major phospholipids which releases DAG for galactolipid synthesis and P. ● Maintains P supply in P- ● Also involved in root elongation in P-	*pldζ1 pldζ2* in P-: decrease in primary root elongation *Pldζ2* in P-*:* decreased PA accumulation	*At*: [170–172]
*SULFOQUINO-VOSYLDIACYLGLYCEROL2 (SQD2)*	● Involved in the replacement of phospholipids with sulfolipids.	*Sqd2* in P-: ● Altered lipid remodelling and impaired growth	*At:* [98, 216]
*GDPD1*	● Involved in the formation of glycerol-3-phosphate (G3P) from phospholipid products, that can be dephosphorylated to release P.	*Gdpd1* in P-: ● Decreased G3P content, P content and seedling growth.	*At*: [173]

##### Genes for P acquisition: Morphological adaptations, PUE transcription factors and P transporters

Specific genes involved in root architecture are targets for enhanced PUE. Although RSA traits are highly quantitative, a BLAST to the rapeseed reference genome revealed 19 candidate genes related to root growth and genetic responses to low P in Arabidopsis [116]. These genes included *AUXIN-INDUCED IN ROOT CULTURES 12* (*AIR12*) involved in auxin-induced production of lateral roots and *PHOSPHATE DEFICICENCY RESPONSE 2* (*PDR2*) which is part of growth changes in the plant apical meristem under P deficiency [137, 138]. *PDR2* is a major component of the P starvation response and functions together with LPR1 and its close paralog LPR2 as a P-sensitive checkpoint in root development by monitoring environmental P concentration, altering meristematic activity and adjusting RSA [139, 140].

**Figure 3:**
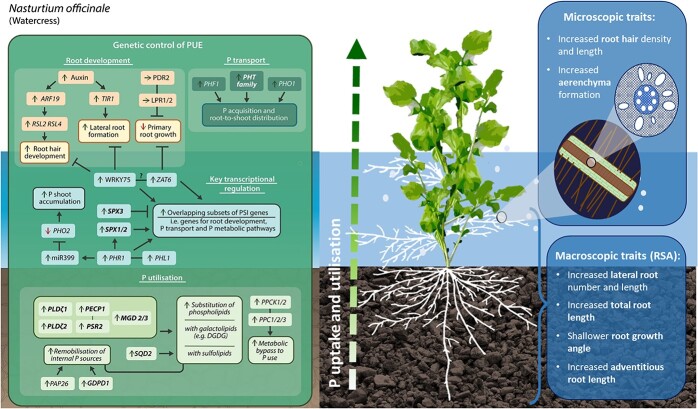
**The watercress PUE ideotype.** Macroscopic and microscopic traits to target for breeding for PUE in watercress are outlined in blue boxes. The green box demonstrates interactions of key PUE genes (identified in *Arabidopsis*) that should be pursued as potential candidate genes for PUE in watercress. Genes in bold are major components of the P response whose expression is induced >10 fold in at least 2 independent Arabidopsis studies, as found by Lan *et al*. (2015). A green up arrow accompanying a gene represents an increase in transcript abundance under P deficiency and a red down arrow indicates a decrease in abundance. Black arrows indicate positive regulation and flat-ended arrows indicate negative regulation under P deficiency. Straight solid black lines represent genes with partial/complete functional redundancy. The question mark shows a hypothetical interaction.

Genes involved in transcriptional control are multi-functional under P deprivation; some have overlapping roles in RSA development, P signalling and P utilisation. They are discussed together here despite partial involvement in P utilisation. *PHR1 (PHOSPHATE STARVATION RESPONSE 1*) and *PHL1* (*PHR-LIKE 1*) code for transcription factors that play critical roles in the control of P starvation responses [141]. PHR1 mediates expression of the microRNA miR399 which modulates the *PHO2* gene, responsible for P allocation between roots and shoots and affects expression of other PSR genes such as PHT transporters [142]. SPX transcription factors (SPX1, SPX2, SPX3, SPX4) are important negative regulators of PSR via repression of *PHR* [143–145]. The roles of several other transcription factor genes on RSA (such as *ZAT6, WRKY75, BHLH32* and *OsPTF1*) and other regulatory elements (such as *SIZ1* and *ARP6*) are summarised in Table 2 and Figure 3.

Auxin, sugars and other hormones such as cytokinins, ethylene, abscisic acid (ABA), giberellins and strigolactones are implicated in phosphate-induced determination of RSA so genes involved in these pathways may be significant candidates [103, 146, 147]. Under low P, auxin levels increase in root hair zones and root tips. Auxin mutants such as *taa1* (involved in auxin synthesis) and *aux1* (involved in auxin transport) have impaired root hair growth in low P [148]. Expression of the Arabidopsis auxin receptor gene *TIR1* increases under low P availability which results in increased sensitivity to auxin and production of lateral roots [149]. Mutants in auxin-inducible transcription factors (e.g. ARF19, OsARF16, OsARF12, RSL2, RSL4) also have disrupted root hair responses under low P. *ROOT HAIR DEFECTIVE 6-LIKE-2* (*RSL2*) and *ROOT HAIR DEFECTIVE 6-LIKE-4* (*RSL4*) are responsive to P deficiency and promote root hair initiation and elongation. *ARF19* is a key transcription factor promoting auxin-dependent root hair elongation in response to low P [148]. *HPS1* (*HYPERSENSITIVE TO PHOSPHATE STARVATION 1*) is involved in regulating the sucrose transporter *SUC2* and *hps1* mutants exhibit significant P-starvation responses under P-sufficient conditions [146]. Plants with impaired cytokinin receptors CRE1 and AHK3 show increased sugar sensitivity and increased expression of P-starvation genes [150]. ETHYLENE RESPONSE FACTOR070 (ERF070) is a transcription factor critical for root development under P starvation. Though no studies exist for P-associated gene expression changes in watercress, Müller *et al*. (2021) used RNA sequencing (RNA-seq) approaches to identify responses to submergence in watercress and found several ABA biosynthesis and catabolism genes associated with stem elongation [47]. This study provides a model for using transcriptomic approaches to explore hormone-induced morphological changes in watercress.

For P acquisition, the *PHT* gene family controlling P transport provides several candidate genes. In Arabidopsis*,* the *PHT* (*PHOSPHATE TRANSPORTER*) genes that encode phosphate transporters responsible for transport of P anions are well characterised and are grouped into four families (*PHT1, PHT2, PHT3* and *PHT4*). PHT proteins other than PHT1 are involved in the uptake, distribution and remobilisation of P within the plant, however, PHT1 in the plasma membrane is the most important [151–153]. Phosphate stress induces expression of these genes. However, the use of PHT transporters in plant breeding has been limited by P toxicity and other side effects of unbalanced P regulation associated with the overexpression of some transporter genes [154]. For example, *OsPHT1;9* and *OsPHT1;10* overexpressing rice plants have reduced biomass under high phosphate compared to wild-type plants [87]. Accessory proteins, encoded for by genes like *PHOSPHATE TRANSPORTER TRAFFIC FACILITATOR* (*PHF1*), are also important for proper functioning of P transporter genes. Homologs of *PHT1* transporter genes, transcriptional factors including the *SPX* gene family, and genes involved in RSA determination such as *PDR2* and *LPR1/2* could be candidate genes for improving phosphate acquisition in watercress, but these genes have yet to be identified in aquatic crops.

##### Genes for P utilisation: P re-translocation, signalling and scavenging

The transcriptional regulation of PUE is complex: there is some overlap with genes involved in both phosphate acquisition and phosphate utilisation, such as the global transcriptional regulation by *PHR1* and *PHL1*. Here we target genes primarily involved in utilisation, including those responsible for P transport within the plant, alternate metabolic pathways, and internal P-scavenging. Using an Arabidopsis Affymetrix gene chip, changes in global gene expression have been analysed in response to P deprivation [155]. The expression of 612 genes was induced and 254 genes suppressed including upregulation of phosphate transporters such as *PHT1* genes and *PHO1;H1* (involved in P loading into xylem vessels) [152]. Genes involved in protein biosynthesis were downregulated during deficiency, likely representing P recycling strategies.

PHT transporters also play a role in P utilisation through re-translocation of P within the plant. Five of the 13 maize *PHT1* genes are induced in other tissues such as leaves, anthers, pollen and seeds, suggesting *PHT1* involvement in diverse processes such as root-to-shoot distribution [86]. PHO1 is another central element responsible for P homeostasis and transport. *Pho1* mutants exhibit P deficiency in the shoots due to lack of P loading into the xylem vessels [96, 156].

Phosphatases are important for remobilisation of fixed P. Eleven genes encoding different purple acid phosphatases were reported to be upregulated under P starvation in Arabidopsis [152]. The Arabidopsis genome encodes 29 purple acid phosphatases, some of which are excreted into the soil, such as PAP12 and PAP26 [92, 157]. Only phosphatase activity within the plant is relevant to watercress breeding: in a flowing water system, any P made available around the roots by secreted phosphatases would rapidly wash away. As well as being a major secreted phosphatase, PAP26 is regarded as the predominant intracellular acid phosphatase in Arabidopsis and is upregulated two-fold under P-deficiency [158]*.* PAP26 functions in PUE by scavenging P from intracellular and extracellular P-ester pools, to increase P availability in the plant. Homologs of *PAP26* should be investigated in watercress.

Plants also respond to P starvation by utilising alternative metabolic pathways. Genes involved in lipid metabolism and biosynthesis represent the largest group of core PSI (phosphate starvation inducible) genes in Arabidopsis, demonstrating their importance in PUE under P starvation [133]. Three genes encode “plant-type” PEPC enzymes in Arabidopsis (*PPC1, PPC2, PPC3*)*. PPC1* is expressed in roots and flowers, *PPC2* in all organs and *PPC3* in only roots [159, 160]. PEPC activity is affected by phosphorylation by PPCK (PEPC kinase), thus *PPCK1* and *PPCK2* genes are additional important components for P-bypassing [99, 161].

Membrane phospholipids constitute approximately 20% of the total P in the leaves of P-sufficient plants [162]. This represents a large pool of P that can be remobilised. 7% of the P-responsive genes found by Misson *et al.* (2005) were involved in lipid biosynthesis pathways in Arabidopsis. This includes the genes *MGD2* and *MGD3* whose expression changed 11-fold and 48-fold under P deficiency, respectively. These genes encode major enzymes for galactolipid biosynthesis and are involved in replacing phospholipids in cell membranes with non-phosphorus lipids such as galactolipid digalactosyldiacylglycerol (DGDG) [163]. *PECP1* is involved in the liberation of P from phospholipids and is upregulated under P deprivation, with up to 1785-fold increases in expression reported in roots [164–166]. *PSR2* encodes a phosphatase involved in galactolipid biosynthesis and whose expression increases 174-fold in P deprived seedlings [167]. Both *PECP1* and *PSR2* have similar roles in the dephosphorylation of phosphocholine (PCho) in the galactolipid synthesis pathway. However, despite their massive upregulation, it has been observed that inactivation of *PECP1* and *PSR2* does not alter plant growth or plant P content under P-deprivation so PCho is not likely a major source of P under limiting conditions [168, 169]. *PLDζ1* and *PLDζ2* encode phospholipases D zeta 1 and 2 that hydrolyse major phospholipids such as phosphatidylcholine which yields phosphatidic acid (PA) and PA phosphatase (PAP) and releases DAG (for galactolipid synthesis) and P [170–172]. Phospholipids can also be replaced by sulfolipids. *SQD2* is the primary gene in this pathway and encodes an enzyme that catalyses the final step in the sulfolipid biosynthesis [98]. *GDPD1* is involved in the formation of glycerol-3-phosphate (G3P) from phospholipid products (such as glycerolphosphoglycerol), that can be dephosphorylated to release P [173].

Homologs of *PHT1* genes responsible for P redistribution, within the plant, genes involved in P scavenging (i.e. *PAP26*), genes implemented in metabolic pathways that bypass P use including galactolipid biosynthetic pathways (e.g. *MGD2/3, PLDζ1/2, PECP1* and *PSR2*) and those involved in sulfolipid biosynthesis (e.g. SQD2) could be candidate genes for improving phosphate utilisation in watercress.

#### Status of watercress breeding

Watercress root research is virtually completely absent in the literature, with no studies on root responses to phosphate availability. Nevertheless, the finite nature of rock phosphate and the fact that watercress cultivation methods have the potential to result in environmental damage (associated with fertiliser input to waterways), are clear drivers, as with soil-grown crops, to breed for watercress with improved PUE. Uncovering phenotypic traits and the molecular basis for PUE are important early steps in breeding for phosphate use efficiency.

No commercial breeding programs exist for watercress worldwide, but germplasm collections are emerging [217–219]. Development of new watercress varieties is also no doubt limited by the lack of genomic information for this crop. Payne (2011) screened a germplasm collection under indoor and outdoor conditions and identified significant variation in several traits including stem length, stem diameter and antioxidant concentrations [218]. Voutsina (2017) used RNA-seq to analyse the first watercress transcriptome and identified differences in antioxidant capacity and glucosinolate biosynthesis across a germplasm collection of watercress, with 71% of the watercress transcripts annotated based on orthology to Arabidopsis [220]. Jeon *et al*. (2017) independently used RNA-seq approaches to assemble the watercress transcriptome *de novo*. They identified 33 candidate genes related to glucosinolate biosynthetic pathways using Arabidopsis glucosinolate genes to search for homologous sequences in the watercress transcriptome [221].

Additionally, a watercress mapping population comprising 259 F_2_ individuals was established by Voutsina (2017) using parents with contrasting nutrient and growth phenotypes. Genotype-by-sequencing of this mapping population enabled the construction of first genetic linkage map for watercress and identified 17 QTL for morphological traits of interest, antioxidant capacity and cytotoxicity against human cancer cells [220]. However, no root traits were assessed in this work.

Screening this mapping population and wider germplasm for root traits may reveal individuals with extreme PUE phenotypes, which could allow the development of markers and QTL associated with this complex trait. RNA-seq approaches could ultimately lead to the identification of candidate genes. Together these findings will assist in developing commercial cultivars with a reduced need for phosphate, and a reduced negative environmental impact. 

To assess whether homologs of the candidate PUE genes identified in this study exist in watercress, available watercress transcriptome data was mined for 13 key PUE genes selected from Table 2. This included genes whose expression was induced more than tenfold in at least 2 independent studies (as found by (Lan *et al*., 2015), plus *PHR1*, the global regulator of P starvation responses. Annotated transcripts were obtained from transcriptomic studies of watercress by Voutsina *et al*., 2016; Jeon *et al*., 2017; Müller *et al*., 2021 and matches for these candidate genes were assessed by searching for genes using AGI (Arabidopsis Gene Identifiers) [47, 133, 221, 222]. Across all studies, strong matches were found for *PHT1;4, SPX1, PHR1, MDG3, PEPC1, PLDζ1/2, PSR2* and *SQD2*, with all corresponding e-values ranging from 0 to 3.00E-32. Additionally, Müller *et al*. identified homologous transcripts for *MDG2*. Voutsina *et al*. had transcripts corresponding to *PHT1;3*, and *MDG2*, and Jeon *et al*. had hits for *PHT1;2* and *PHT1;3*. No matching transcripts were found for *PHT1;1* in any of the three studies. Where FDR values (p-values) were < 0.05, changes to expression patterns were noted. Interestingly, Müller *et al*. also observed varying levels of upregulation of *PHT1;4* and *PHR1* following submergence, which may suggest a link between phosphate starvation and submergence responses.

## Conclusions

Watercress is a non-model leafy green crop, grown using traditional aquatic systems across the world, but also increasingly seen as a suitable crop for vertical hydroponic indoor systems. It is ranked as the top “powerhouse” food, with the highest nutrient density, including high concentrations of essential vitamins, minerals and phytonutrients [223]. However, regulation from environmental agencies on nutrient inputs to water systems is increasing the pressure to reduce fertiliser use in traditional aquatic growing systems [38, 224].

Breeding new varieties with improved phosphate use efficiency (PUE) is thus a priority for both traditional outdoor and indoor systems. Prebreeding for PUE in aquatic crops such as watercress should target a suite of traits (Fig. 3), including (i) increased total root length, (ii) number of root tips, (iii) lateral and adventitious root number and length as important root architectural traits. Microscopic traits of interest include (iv) increased aerenchyma formation, (v) increased root hair density and length. At the genomic level, (vi) enhanced activity of P transporter genes (e.g. *PHT* homologs) and (vii) genes involved in P utilisation, particularly those with roles in P remobilisation (e.g. *PAP26*, *MGD3, PEPC1*) and P-bypass enzymes (e.g. *PPC1*), may prove good candidate genes for PUE.

Barriers for root trait breeding include the difficulties in evaluating roots in the substrate, their phenotypic plasticity in response to numerous environmental factors and limited genomic knowledge for watercress. Recent developments in high-throughput phenotyping and whole-genome sequencing-based genotyping will accelerate QTL identification, the discovery of individual genes involved in PUE and lay the foundations for development of new watercress varieties with improved low P tolerance.
